# Estimation of Heart Rate and Energy Expenditure Using a Smart Bracelet during Different Exercise Intensities: A Reliability and Validity Study

**DOI:** 10.3390/s22134661

**Published:** 2022-06-21

**Authors:** Yihui Cai, Zi Wang, Wanxia Zhang, Weiya Kong, Jiayao Jiang, Ruobing Zhao, Dongxue Wang, Leyi Feng, Guoxin Ni

**Affiliations:** 1 School of Sports Medicine and Rehabilitation, Beijing Sport University, Beijing 100084, China; 2020210445@bsu.edu.cn (Y.C.); 2020210408@bsu.edu.cn (Z.W.); 2019210446@bsu.edu.cn (W.Z.); 2020012006@bsu.edu.cn (W.K.); rehab_jiang@bsu.edu.cn (J.J.); 2021210079@bsu.edu.cn (R.Z.); 2021210043@bsu.edu.cn (D.W.); 2 School of Sport Science, Beijing Sport University, Beijing 100084, China; 2019210154@bsu.edu.cn

**Keywords:** exercise, wrist-worn devices, monitoring, energy expenditure, heart rate, reliability, validation

## Abstract

Background. With wrist-worn wearables becoming increasingly available, it is important to understand their reliability and validity in different conditions. The primary objective of this study was to examine the reliability and validity of the Lexin Mio smart bracelet in measuring heart rate (HR) and energy expenditure (EE) in people with different physical activity levels exercising at different intensities. Methods. A total of 65 participants completed one maximal oxygen uptake test and two running exercise tests wearing the Mio smart bracelet, the Polar H10 HR band, and a gas-analysis system. Results. In terms of HR measurement reliability, the Mio smart bracelet showed good reliability in a left versus right test and good test–retest reliability (*p* > 0.05; mean absolute percentage error (MAPE) < 10%; intraclass correlation coefficient (ICC) > 0.4). For EE measurement, the Mio smart bracelet showed good reliability in a left versus right test, good test–retest reliability on the right (*p* > 0.05; MAPE > 10%; ICC > 0.4), and low test–retest reliability on the left (*p* > 0.05; MAPE > 10%; ICC < 0.4). Regarding validity, the Mio smart bracelet showed good validity for HR measurement (*p* > 0.05; MAPE < 10%; ICC > 0.4) and low validity for EE measurement (*p* < 0.05; MAPE > 10%; ICC < 0.4). Conclusion. The Lexin Mio smart bracelet showed good reliability and validity for HR measurement among people with different physical activity levels exercising at various exercise intensities in a laboratory setting. However, the smart bracelet showed good reliability and low validity for the estimation of EE.

## 1. Introduction

According to the physical activity guidelines of the American College of Sports Medicine (ACSM), adults should receive health benefits when they undertake 150 min of moderate-intensity cardiorespiratory exercise training or 75 min of vigorous-intensity cardiorespiratory exercise training per week [1,2,3]. To maximize the benefits gained from exercise, it is essential for people to monitor their heart rate (HR) and energy expenditure (EE) during exercise. HR monitoring makes it possible for people to track their exercise intensity and quantify it in a valuable way, and EE monitoring could help people better control the time and volume of exercise.

Electrocardiography (ECG) and Polar HR monitors are generally used for HR monitoring [4,5,6]. However, ECG is more suitable for laboratory and hospital settings than for individual use, and wearing a Polar HR chest strap involves a very complex process. Measuring EE relies on the use of different methods, with the indirect calorimetry and double-labeled water methods recognized as the “gold standards” [7,8,9]. However, their high cost, large equipment size, complex processes, cumbersome analysis, and data lag make them suitable for use only in a laboratory or for small groups.

Today, wearable devices, represented by sports bracelets, are rapidly gaining popularity worldwide due to their low cost, ease of wear, low activity restrictions, and ability to provide simple and timely data on some physical activity indicators (e.g., HR, EE, and step count). In one American study, approximately 62% of adults reported that they met the ACSM’s physical activity guidelines; however, when their physical activity was monitored using an accelerometer, the percentage of those meeting the guidelines was found to be 9.6% lower [10]. Thus, wearable devices that monitor exercise, such as accelerometers, can provide accurate information on physical activity, which is key to both the development of effective exercise intervention strategies and the improvement of people’s health [11].

As wearable devices become more common, it is critical that they produce reliable and valid data. In terms of HR measurement, many peer-reviewed studies have been conducted to evaluate the reliability and validity of wearable devices produced by different brands, such as Fizzo, Fitbit, Apple Watch, and Garmin [6,12,13,14,15,16,17]. Some studies have shown that these devices more accurately measure HR at lower treadmill speeds [18,19,20]; however, others have shown that they are more accurate at higher treadmill speeds [21]. For the measurement of EE, most studies have revealed that wearable devices return inaccurate measurements [12,16,17,22]. Shcherbina et al. [16] tested seven devices on healthy subjects and found that while HR measurements were within acceptable margins of error, none of the tested devices’ EE measurements were within acceptable ranges. The limitations of that study include the fact that different devices and exercise regimens were used, different exercise intensities were tested, and different reference standards were used. Due to these limitations, the reliability and validity of the findings cannot be generalized to all wearable devices. Moreover, considering the individual differences within large populations, wearable devices should have good reliability and validity for people with different physical activity levels and different exercise intensities. However, no previous study has evaluated the measurement accuracy of wearable devices in individuals with different physical activity levels and different exercise intensities.

Some previous studies compared the measurement results of wearable devices when they are worn on the dominant and non-dominant hands. However, the findings of these studies are inconsistent [6,23,24]. Considering the accelerating rate of technological change and the increasing speed at which wearable devices are being updated, it is important to evaluate the reliability and validity of current devices, especially those that are popular. In this study, we evaluated the new and popular Lexin Mio smart bracelet (Mio Band 1) due to its reasonable cost and compatibility with both Android and iOS systems. Previous studies on Mio smart bracelets have been sparse and based primarily on the accuracy of predicted EE [22]. The manufacturer of the device used in the present study does not specify whether the smart bracelet should be worn on the dominant or non-dominant wrist or whether different results should be expected based on the wrist type.

Therefore, the first objective of this study was to test the reliability and validity of a popular wearable activity monitor in assessing the HR and EE of people with different physical activity levels exercising at different intensities. The second objective was to determine whether there was a difference in the physical activity assessment when the device was worn on the dominant wrist and non-dominant wrist. This was a preliminary study conducted in a laboratory environment to simplify data recording and exclude unexpected perturbations. Based on the outcomes of this study, future studies may be conducted in a real-world environment.

Based on the findings of previous studies, we hypothesized that the smart bracelet has good reliability and validity in measuring HR and that the EE measurement results are inferior to the HR measurement results. In addition, we hypothesized that wearing the smart bracelet on the dominant or non-dominant hand does not affect the effectiveness of the physical activity assessment.

## 2. Materials and Methods

### 2.1. Ethics

The study was conducted according to the guidelines of the Declaration of Helsinki and approved by the Experimental Ethics Committee for Sports Science of Beijing Sport University (Approval number: 2020133H, 16 September 2021). Informed consent was obtained from all subjects involved in the study.

### 2.2. Participants

The number of participants was set at 65 (more than 20 people per group) based on the findings of Wallen et al. [25], who used a power of 0.5 and a type I error probability of 5%. This sample size is comparable to that of previous studies [6,16,26] that examined the use of wrist-worn health-tracking devices, which included anywhere from 20 to 60 individuals. All of the participants were healthy, right-handed students (41 females and 24 males) aged 18–38 years (mean = 22.06 years) from Beijing Sport University. They were selected based on the following inclusion criteria: (a) aged 18–40 years; (b) no contraindications to exercise; and (c) not concurrently participating in another biomedical study. Some previous articles have shown that skin tone affects the HR readings of optical sensors [27]. To avoid the effect of skin tone, all of the subjects recruited for this study were Chinese.

Before each test, the participants were prohibited from consuming alcohol or caffeine for 12 h and from eating anything for >1 h. They were reminded to wear comfortable sportswear and shoes during the test. If a participant had not complied with these conditions, their test was rescheduled. Before the first test, each participant was asked to complete an informed consent form, the Physical Activity Readiness Questionnaires (PAR-Q), and the International Physical Activity Questionnaire (IPAQ). The IPAQ is one of the most valid and widely used questionnaires for measuring physical activity levels in adults aged 15–69 years and has been used in Chinese population studies [28,29,30]. Each participant’s physical activity level was calculated using the IPAQ results, and the results were used to group the participants into three categories: those with a low physical activity level (LPAL, *n* = 22), those with a moderate physical activity level (MPAL, *n* = 23), and those with a high physical activity level (HPAL, *n* = 20). When each participant completed the entire test, they were given a personalized aerobic exercise prescription.

### 2.3. Wearable Devices

**Figure 1:**
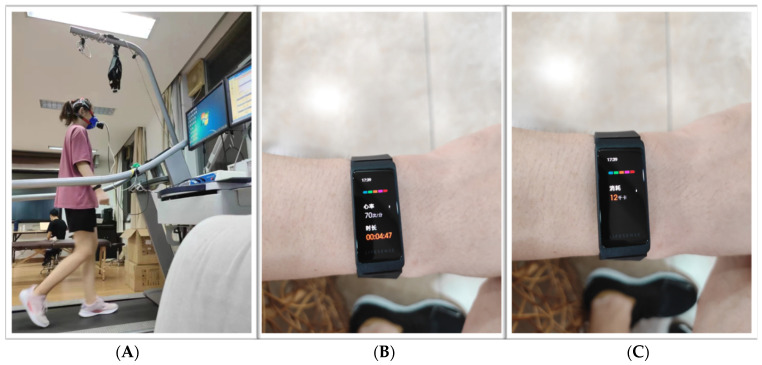
A subject walked on a treadmill with one bracelet on each wrist (**A**), monitoring heart rate (**B**) and energy expenditure (**C**), respectively.

During the test, the participants wore two wearable motion monitors at the same time: a Mio smart bracelet on each wrist (henceforth, “left Mio” and “right Mio”) (Figure 1).

The Mio smart bracelet is a wristband wearable device made in China and based on photoplethysmography (PPG) and a three-axis acceleration sensor. This device provides multidimensional health indicator data on HR, EE, mileage, step count, sleep length, stress level, and blood oxygen saturation.

In terms of HR, the basis for measuring HR is PPG, an optical technique in which HR is determined by monitoring changes in blood volume beneath the skin [31]. The HR sensor collects dynamic HR data at a rate of once per second and displays the HR on the active-matrix organic light-emitting diode (AMOLED) screen of the bracelet in real-time. In addition, an exercise HR range and a high HR warning value can be set through a mobile phone application, as well as an alert for when the HR is too high to ensure the safety of the wearer. EE is calculated using algorithms that are not openly disclosed [25].

### 2.4. Criterion Measures 

In addition to the Mio smart bracelets, the participants wore the following standard measurement devices to examine the accuracy of the Mio smart bracelets. 

#### 2.4.1. Polar Heart Rate Monitor

A Polar H10 HR monitor (Polar Electro, Helsinki, Finland) was used as the standard instrument for measuring HR along with the Mio smart bracelet, as studies have shown it to be a valid and reliable tool for HR measurement compared to the 12-lead ECG [4,5,6]. An HR sensor is worn on the chest and transmits the user’s real-time HR data to a watch.

#### 2.4.2. Cardiorespiratory Function Test System

EE was measured using a benchtop gas-analysis system (GAS, Metalyzer 3B, Cortex, Leipzig, Germany) and indirect calorimetry. The subject’s inhaled and exhaled gases were collected through a gas collection tube (Hans Rudolph pneumotachometer) connected to a face mask and then analyzed using the GAS. The following parameters were measured: HR, total EE (kcal), oxygen consumption (VO_2_), carbon dioxide production (VCO_2_), and respiratory quotient (RQ). The Weir equation [32] was used: EE (kcal/min) = 3.9 VO_2_ (L/min) + 1.1 VCO_2_ (L/min). Before each test, the instrument was warmed up for 30–40 min, and then separate calibrations were performed, including room air calibration, standard gas calibration, capacity calibration, and delayed calibration. After all the calibrations were performed, the instrument was officially tested. The GAS used in this experiment has been proven to be a reliable instrument for testing EE in sports medicine research [33].

### 2.5. Other Measures

Each participant’s height and weight were measured. They were instructed to wear sports attire and were asked to remove their shoes before having their height and weight measured twice. When there was a difference of 0.25 cm in height or 0.1 kg in weight between the two measurements, a third measurement was taken. The participant’s height and weight were entered into the Mio smart bracelet and GAS before the exercise test began. The Borg Rating of Perceived Exertion (RPE) scale was used to measure the participants’ perceived level of exercise fatigue while they performed the test. The scores ranged from 6 to 20, which corresponded to HRs of 60–200 beats per minute (bpm) [34]. 

### 2.6. Procedures

**Figure 2:**
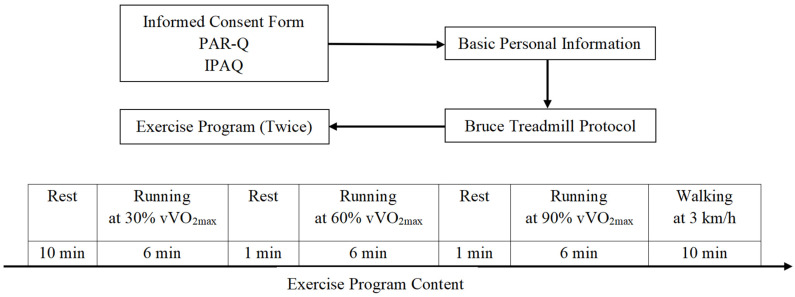
The study procedure. PAR-Q: Physical Activity Readiness Questionnaires; IPAQ: International Physical Activity Questionnaire; vVO_2_max: Speed at maximum oxygen uptake.

The study procedure is shown in Figure 2. After completing the informed consent form, the PAR-Q, and the IPAQ, the participants had their basic personal measurements recorded (i.e., height, weight, waist circumference, hip circumference, and blood pressure). Trained master’s students performed the anthropometric measurements.

Each participant’s measurements were used to initialize the wearable device and the GAS. Two Mio smart bracelets were placed on the left and right wrists, above the ulnar styloid process. At the same time, the Polar HR band was placed at the level of the participant’s thoracic glabella with the help of the researcher. All three devices were tightly secured to ensure contact with the skin [35], and then the participant was fitted with a breathing mask and the GAS was connected to the breathing mask.

The laboratory temperature was maintained at 25 ± 1 °C, the relative humidity was 40–60%, and the laboratory environment was quiet and free of noise. To avoid unexpected problems with the functionality of the Mio device, 20 Mio smart bracelets were available.

The test was conducted three times for each participant. The first test was mainly used to measure basic anthropometric data and maximum oxygen uptake (VO_2_max). This test was performed according to the Bruce treadmill protocol, and VO_2_max was assessed with the GAS. The standard Bruce Protocol was utilized, with increases in speed (Stage 1, 2.7 km/h; Stage 2, 4.0 km/h; Stage 3, 5.4 km/h; Stage 4, 6.7 km/h; and Stage 5, 8.0 km/h) and incline (starting at 10% grade and increasing 2% at every stage) every 3 min until exhaustion [36]. When participants met two of the following four requirements, the test was terminated: (1) a plateau in HR despite increased activity; (2) peak oxygen uptake during incremental exercise, with constant or small increases in oxygen uptake as the load increases; (3) failure to keep up with treadmill speed; and (4) respiratory exchange ratio (RER) > 1.10. On a subjective level, exercise testing could also be terminated when the subject reached volitional exhaustion (RPE of 19 or 20) [37,38]. The second and third tests were mainly used to measure HR and EE during an exercise program. These two tests required the participants to remain in a quiet, sitting position for 10 min. Then, the participants completed the running test at 30% vVO_2_max, 60% vVO_2_max, and 90% vVO_2_max, which corresponded to three speeds on a 1% running platform slope. The expected intensity × VO_2_max = 3.5 + (0.2 × vVO_2_max) + (0.9 × vVO_2_max × slope%) [39]. We used the 1% slope to simulate the air resistance that runners experience outdoors [40]. The test time for each exercise intensity was 6 min, with a 1-min interval between intensities. The last stage was a 10-min recovery period. The RPE was measured in the last 15 s of each phase. The latter two tests needed to be conducted at the same time on different days, and the interval between the three tests was 48 h to 5 days.

If a participant was uncomfortable during any of the tests, the treadmill emergency device could be used to immediately stop the treadmill. A trained researcher was present beside the treadmill for every test to adjust the speed and protect the participants.

### 2.7. Data Processing

The raw HR data were obtained by accessing the backend website of the Mio smart bracelet (https://jiankang.coolplay.tv/users/index, accessed on 20 November 2021) and were exported to Excel. The required data were extracted and transferred to Statistical Product Service Solutions (SPSS) for analysis. The second and third tests used the Mio smart bracelet, the Polar HR monitor, and the GAS to simultaneously monitor the participants’ HR and EE in real-time. To allow the participants sufficient time to reach a steady state, the average HR and EE values were recorded during the last 4 min of the rest periods, during the middle 4 min of the three exercise intensity running phases, and during the entire recovery period.

### 2.8. Statistical Analyses

SPSS statistical software (Version 26; SPSS, IBM Corporation, Armonk, NY, USA) was used to analyze the HR and EE data collected by each device. Descriptive data were reported as mean and standard deviation values. Reliability was determined by measuring the left–right Mios’ consistency and ipsilateral measurement repeatability. The left–right Mios’ consistencies were calculated using the paired Student’s *t*-test (*t*-test), mean absolute percentage error (MAPE), Pearson’s correlation coefficient, and intraclass correlation coefficient (ICC) between the left and right Mio data. Ipsilateral measurement repeatability was calculated using the *t*-test, MAPE, Pearson’s correlation coefficient, and ICC between two measurements on one side.

To determine validity, the *t*-test, MAPE, Pearson’s correlation coefficient, ICC, and Bland–Altman statistical methods were used between the left and right Mios and the standard measurement devices (Polar and GAS).

In the *t*-test, a *p*-value ≥ 0.05 indicated no difference in the data and good reliability/validity. The MAPE was used to assess the degree of error between the standard measurement instrument output and the Mio smart bracelet output for each measured value. A MAPE < 5% indicated high reliability/validity (precise), 5% ≤ MAPE ≤ 10% was acceptable, and a MAPE > 10% indicated low reliability/validity (imprecise) [16,41,42]. The Pearson’s correlation was calculated to determine the degree of correlation between mean values, and a *p* < 0.05 was statistically significant; the closer the correlation coefficient was to 1, the more correlated it was, indicating good reliability/validity. For the ICC, absolute agreement in a two-way mixed effect model was analyzed. The ICC was interpreted as low (<0.4), moderate (0.4–0.75), or high (>0.75) [43]. For the Bland–Altman statistical methods, the bias (mean value of the difference (MD)) and the limits of agreement (MD ± 1.96 × standard deviation of the mean difference (LOA)) were analyzed. If the difference between the two measurements was within 95% LOA, the two devices could be considered in good agreement (the more focused the scatter plot trend, the better). In general, if more than half of the results were acceptable, they were considered to have good reliability/validity.

## 3. Results

### 3.1. Descriptive Statistics

A total of 65 individuals completed the study, 63% of whom were female (*n* = 41) and 37% of whom were male (*n* = 24). Their physical activity levels were as follows: 22 had a LPAL (*n* = 22), 23 had a MPAL (*n* = 23), and 20 had a HPAL (*n* = 20). Table 1 shows the descriptive statistics for the participant characteristics, and Table 2 and Table 3 show the descriptive statistics for the participants’ HR and EE data, respectively.

### 3.2. Reliability

#### 3.2.1. Heart Rate

Table 4 shows the reliability in the left Mio versus right Mio test and the test–retest reliability for HR measurements.

In the LPAL group, the *p* value of the *t*-test ranged from 0.048 to 0.932 (*p* > 0.05, except for the right Mio at high intensity), the MAPE ranged from 0.5% to 4.65% (MAPE < 5%), the Pearson’s correlation coefficients ranged from 0.448 to 0.997 (*p* < 0.05), and the ICC ranged from 0.448 to 0.997 (ICC > 0.4).

In the MPAL group, the *p* value of the *t*-test ranged from 0.169 to 1 (*p* > 0.05), the MAPE ranged from 0.61% to 6.32% (MAPE < 10%), the Pearson’s correlation coefficients ranged from 0.378 to 0.998 (*p* < 0.05, except for the right Mio at low intensity), and the ICC ranged from 0.376 to 0.998 (ICC > 0.4, except for the right Mio at low intensity).

In the HPAL group, the *p* value of the *t*-test ranged from 0.097 to 0.97 (*p* > 0.05), the MAPE ranged from 1.29% to 6.94% (MAPE < 10%), the Pearson’s correlation coefficients ranged from 0.124 to 0.992 (*p* < 0.05, except for the left versus right Mio at high intensity, the left Mio at baseline, and the right Mio at high intensity), and the ICC ranged from 0.111 to 0.992 (ICC > 0.4, except for the left versus right Mio at high intensity, the left Mio at baseline, and the right Mio at high intensity).

Overall, the Mio smart bracelet showed good reliability in the left versus right Mio test and good test–retest reliability for HR measurement. However, as the level of physical activity increased, its reliability decreased slightly: the reliability was relatively poor at high intensity, and the reliability of the right Mio was relatively poor.

#### 3.2.2. Energy Expenditure

Table 5 shows the reliability in the left versus right Mio test and test–retest reliability for EE measurement.

In the LPAL group, the *p* value of the *t*-test ranged from 0.104 to 0.943 (*p* > 0.05), the MAPE ranged from 16.26% to 20.68% (MAPE > 10%), the Pearson’s correlation coefficients ranged from 0.025 to 0.887 (*p* < 0.05, except for the left versus right Mio at high intensity and the retest measurements of the left Mio), and the ICC ranged from 0.02 to 0.879 (ICC > 0.4, except for the left versus right Mio at high intensity and the retest measurements of the left Mio).

In the MPAL groups, the *p* value of the *t*-test ranged from 0.048 to 0.895 (*p* > 0.05, except for the left Mio at high intensity), the MAPE ranged from 13.99% to 21.82% (MAPE > 10%), the Pearson’s correlation coefficients ranged from 0.242 to 0.813 (*p* < 0.05, except for the left Mio at baseline, low intensity, and moderate intensity), and the ICC ranged from 0.241 to 0.812 (ICC > 0.4, except for the left Mio at baseline, low intensity, and moderate intensity).

In the HPAL group, the *p* value of the *t*-test ranged from 0.017 to 0.998 (*p* > 0.05, except for the left versus right Mio at baseline), the MAPE ranged from 21.01% to 30.63% (MAPE > 10%), the Pearson’s correlation coefficients ranged from 0.046 to 0.557 (*p* < 0.05, except for the left versus right Mio at low intensity and high intensity, the retest measurements of the left Mio, and the right Mio at baseline, low intensity, high intensity, and recovery), and the ICC ranged from 0.043 to 0.556 (ICC > 0.4, except for the left versus right Mio at low intensity and high intensity, the retest measurements of the left Mio, and the right Mio at baseline, high intensity, and recovery).

In general, the Mio smart bracelet showed good reliability in the left versus right Mio test and good test–retest reliability of the right Mio for EE measurement. The test–retest reliability of the left Mio was poor; as the level of physical activity increased, its reliability decreased, and the reliability was relatively poor at high intensity.

### 3.3. Validity

#### 3.3.1. Heart Rate

**Figure 3:**
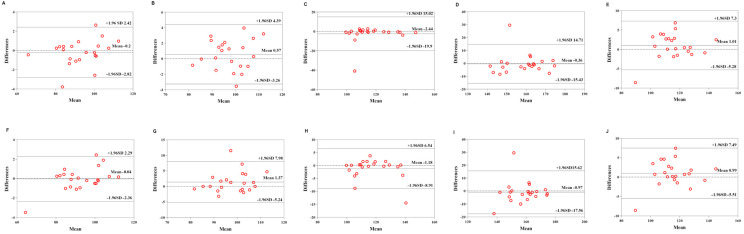
Comparison between the Lexin Mio smart bracelet (Mio) and Polar Heart Rate Monitor (Polar) for heart rate (HR) in the low-physical-activity level (LPAL) group. (**A**) Bland–Altman plots of mean of left Mio and Polar for HR under rest condition. (**B**) Bland–Altman plots of mean of left Mio and Polar for HR under the low-intensity exercise. (**C**) Bland–Altman plots of mean of left Mio and Polar for HR under the middle-intensity exercise. (**D**) Bland–Altman plots of mean of left Mio and Polar for HR under the high-intensity exercise. (**E**) Bland–Altman plots of mean of left Mio and Polar for HR under recovery condition. (**F**) Bland–Altman plots of mean of right Mio and Polar for HR under rest condition. (**G**) Bland–Altman plots of mean of right Mio and Polar for HR under the low-intensity exercise. (**H**) Bland–Altman plots of mean of right Mio and Polar for HR under the middle-intensity exercise. (**I**) Bland–Altman plots of mean of right Mio and Polar for HR under the high-intensity exercise. (**J**) Bland–Altman plots of mean of right Mio and Polar for HR under recovery condition.

**Figure 4:**
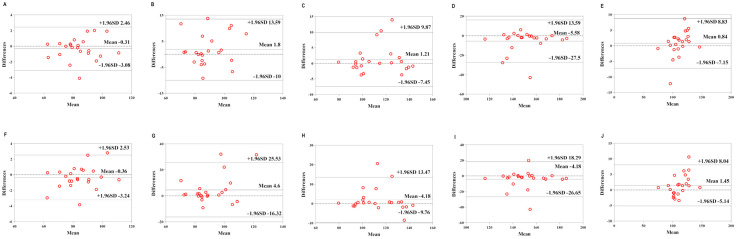
Comparison between the Lexin Mio smart bracelet (Mio) and Polar Heart Rate Monitor (Polar) for heart rate (HR) in the middle-physical-activity level (MPAL) group. (**A**) Bland–Altman plots of mean of left Mio and Polar for HR under rest condition. (**B**) Bland–Altman plots of mean of left Mio and Polar for HR under the low-intensity exercise. (**C**) Bland–Altman plots of mean of left Mio and Polar for HR under the middle-intensity exercise. (**D**) Bland–Altman plots of mean of left Mio and Polar for HR under the high-intensity exercise. (**E**) Bland–Altman plots of mean of left Mio and Polar for HR under recovery condition. (**F**) Bland–Altman plots of mean of right Mio and Polar for HR under rest condition. (**G**) Bland–Altman plots of mean of right Mio and Polar for HR under the low-intensity exercise. (**H**) Bland–Altman plots of mean of right Mio and Polar for HR under the middle-intensity exercise. (**I**) Bland–Altman plots of mean of right Mio and Polar for HR under the high-intensity exercise. (**J**) Bland–Altman plots of mean of right Mio and Polar for HR under recovery condition.

**Figure 5:**
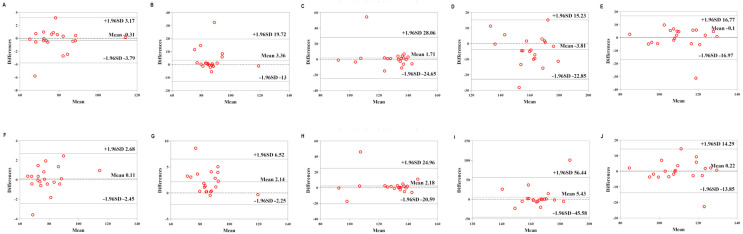
Comparison between the Lexin Mio smart bracelet (Mio) and Polar Heart Rate Monitor (Polar) for heart rate (HR) in the high-physical-activity level (HPAL) group. (**A**) Bland–Altman plots of mean of left Mio and Polar for HR under rest condition. (**B**) Bland–Altman plots of mean of left Mio and Polar for HR under the low-intensity exercise. (**C**) Bland–Altman plots of mean of left Mio and Polar for HR under the middle-intensity exercise. (**D**) Bland–Altman plots of mean of left Mio and Polar for HR under the high-intensity exercise. (**E**) Bland–Altman plots of mean of left Mio and Polar for HR under recovery condition. (**F**) Bland–Altman plots of mean of right Mio and Polar for HR under rest condition. (**G**) Bland–Altman plots of mean of right Mio and Polar for HR under the low-intensity exercise. (**H**) Bland–Altman plots of mean of right Mio and Polar for HR under the middle-intensity exercise. (**I**) Bland–Altman plots of mean of right Mio and Polar for HR under the high-intensity exercise. (**J**) Bland–Altman plots of mean of right Mio and Polar for HR under recovery condition.

Table 6 shows the validity of the Mio smart bracelet versus the Polar H10 HR monitor. The Bland–Altman plots (Figure 3, Figure 4 and Figure 5) all demonstrated that the majority of the values were within LOA.

In the LPAL group, the *p* value of the *t*-test ranged from 0.07 to 0.891 (*p* > 0.05), the MAPE ranged from 1% to 4.22% (MAPE < 5%), the Pearson’s correlation coefficients ranged from 0.704 to 0.996 (*p* < 0.05), and the ICC ranged from 0.702 to 0.994 (ICC > 0.4).

In the MPAL group, the *p* value of the *t*-test ranged from 0.026 to 0.333 (*p* > 0.05, except for the left Mio at high intensity), the MAPE ranged from 1.26% to 7.55% (MAPE < 10%), the Pearson’s correlation coefficients ranged from 0.727 to 0.993 (*p* < 0.05), and the ICC ranged from 0.697 to 0.993 (ICC > 0.4).

In the HPAL group, the *p* value of the *t*-test ranged from <0.001 to 0.959 (*p* > 0.05, except for the right Mio at low intensity), the MAPE ranged from 1.16% to 7.05% (MAPE < 10%), the Pearson’s correlation coefficients ranged from 0.622 to 0.994 (*p* < 0.05, except for the right Mio at high intensity), and the ICC ranged from 0.611 to 0.993 (ICC > 0.4, except for the right Mio at high intensity).

Overall, the Mio smart bracelet showed good validity for HR measurement. However, as the level of physical activity increased, its validity decreased slightly; the validity was relatively poor at high intensity.

#### 3.3.2. Energy Expenditure

**Figure 6:**
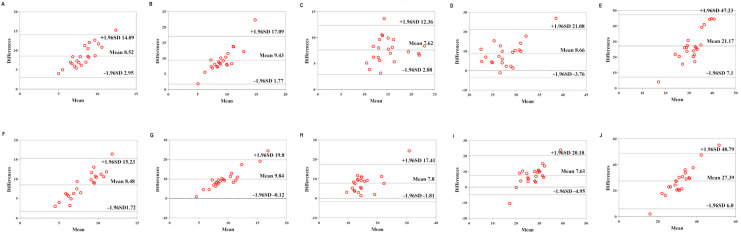
Comparison between the Lexin Mio smart bracelet (Mio) and gas-analysis system (GAS) for energy expenditure (EE) in the low-physical-activity level (LPAL) group. (**A**) Bland–Altman plots of mean of left Mio and GAS for EE under rest condition. (**B**) Bland–Altman plots of mean of left Mio and GAS for EE under the low-intensity exercise. (**C**) Bland–Altman plots of mean of left Mio and GAS for EE under the middle-intensity exercise. (**D**) Bland–Altman plots of mean of left Mio and GAS for EE under the high-intensity exercise. (**E**) Bland–Altman plots of mean of left Mio and GAS for EE under recovery condition. (**F**) Bland–Altman plots of mean of right Mio and GAS for EE under rest condition. (**G**) Bland–Altman plots of mean of right Mio and GAS for EE under the low-intensity exercise. (**H**) Bland–Altman plots of mean of right Mio and GAS for EE under the middle-intensity exercise. (**I**) Bland–Altman plots of mean of right Mio and GAS for EE under the high-intensity exercise. (**J**) Bland–Altman plots of mean of right Mio and GAS for EE under recovery condition.

**Figure 7:**
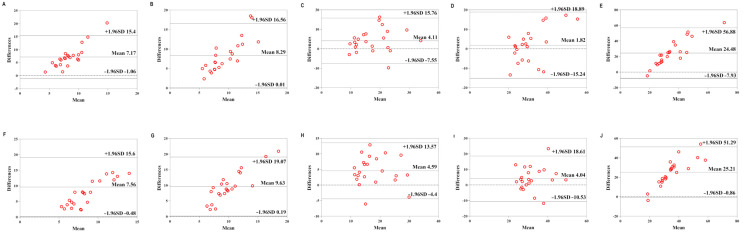
Comparison between the Lexin Mio smart bracelet (Mio) and gas-analysis system (GAS) for energy expenditure (EE) in the middle-physical-activity level (MPAL) group. (**A**) Bland–Altman plots of mean of left Mio and GAS for EE under rest condition. (**B**) Bland–Altman plots of mean of left Mio and GAS for EE under the low-intensity exercise. (**C**) Bland–Altman plots of mean of left Mio and GAS for EE under the middle-intensity exercise. (**D**) Bland–Altman plots of mean of left Mio and GAS for EE under the high-intensity exercise. (**E**) Bland–Altman plots of mean of left Mio and GAS for EE under recovery condition. (**F**) Bland–Altman plots of mean of right Mio and GAS for EE under rest condition. (**G**) Bland–Altman plots of mean of right Mio and GAS for EE under the low-intensity exercise. (**H**) Bland–Altman plots of mean of right Mio and GAS for EE under the middle-intensity exercise. (**I**) Bland–Altman plots of mean of right Mio and GAS for EE under the high-intensity exercise. (**J**) Bland–Altman plots of mean of right Mio and GAS for EE under recovery condition.

**Figure 8:**
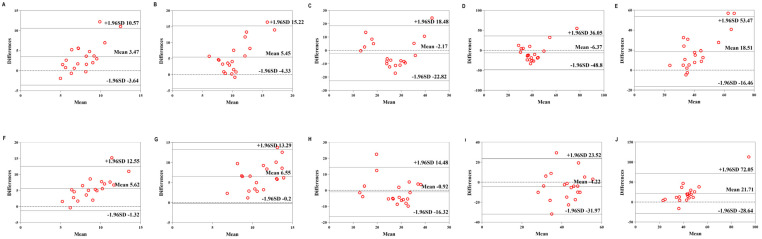
Comparison between the Lexin Mio smart bracelet (Mio) and gas-analysis system (GAS) for energy expenditure (EE) in the high-physical-activity level (HPAL) group. (**A**) Bland–Altman plots of mean of left Mio and GAS for EE under rest condition. (**B**) Bland–Altman plots of mean of left Mio and GAS for EE under the low-intensity exercise. (**C**) Bland–Altman plots of mean of left Mio and GAS for EE under the middle-intensity exercise. (**D**) Bland–Altman plots of mean of left Mio and GAS for EE under the high-intensity exercise. (**E**) Bland–Altman plots of mean of left Mio and GAS for EE under recovery condition. (**F**) Bland–Altman plots of mean of right Mio and GAS for EE under rest condition. (**G**) Bland–Altman plots of mean of right Mio and GAS for EE under the low-intensity exercise. (**H**) Bland–Altman plots of mean of right Mio and GAS for EE under the middle-intensity exercise. (**I**) Bland–Altman plots of mean of right Mio and GAS for EE under the high-intensity exercise. (**J**) Bland–Altman plots of mean of right Mio and GAS for EE under recovery condition.

Table 7 shows the validity of the Mio smart bracelet versus the GAS. The Bland–Altman plots (Figure 6, Figure 7 and Figure 8) all showed that most of the values were within LOA.

In the LPAL group, the *p* values of the *t*-test were all <0.001, the MAPE ranged from 10.37% to 27.52% (MAPE > 10%), the Pearson’s correlation coefficients ranged from 0.111 to 0.782 (*p* < 0.05, except for the left Mio at baseline, low intensity, high intensity, and recovery and the right Mio at low intensity), and the ICC ranged from 0.058 to 0.780 (ICC < 0.4, except for the left Mio at moderate intensity and the right Mio at moderate intensity).

In the MPAL group, the *p* value of the *t*-test ranged from <0.001 to 0.326 (*p* < 0.05, except for the left Mio at high intensity), the MAPE ranged from 16.4% to 32.42% (MAPE > 10%), the Pearson’s correlation coefficients ranged from 0.166 to 0. 739 (*p* < 0.05, except for the left Mio at baseline and the right Mio at baseline), and the ICC ranged from 0.096 to 0.738 (ICC > 0.4, except for the left Mio at baseline, low intensity and recovery and the right Mio at baseline, low intensity, and recovery).

In the HPAL group, the *p* value of the *t*-test ranged from <0.001 to 0.607 (*p* < 0.05, except for the left Mio at moderate intensity and high intensity and the right Mio at moderate intensity and high intensity), the MAPE ranged from 19.64% to 30.33% (MAPE > 10%), the Pearson’s correlation coefficients ranged from 0.067 to 0.583 (*p* > 0.05, except for the right Mio at moderate intensity), and the ICC ranged from 0.067 to 0.571 (ICC < 0.4, except for the left Mio at moderate intensity and the right Mio at moderate intensity). In general, the Mio smart bracelet showed poor validity for EE measurement.

## 4. Discussion

This study examined the reliability and validity of a commonly used wrist-worn, consumer-grade activity monitor for measuring HR and EE at different exercise intensities in people with different physical activity levels. The results of this study are generally consistent with those of previous studies [20,25,35]. Regarding HR measurement, in most cases, this wearable device had good reliability and validity in the laboratory. However, the device performed poorly in measuring EE. This result is also consistent with those of previous studies, in which most wearable devices were found to measure EE with low reliability and validity [12,16,44,45].

In terms of HR monitoring, the reliability and validity of the Mio smart bracelet decreased as the level of physical activity and exercise intensity increased. This was similar to the results of previous studies [20,46]. This may be because the acceleration sensor is not sensitive enough to measure exact values when the treadmill is operating at high speeds.

Regarding EE, it remains unclear why the device was not able to accurately measure this parameter, as the calculation of EE values depends on many anthropometric characteristics derived from the HR [47]. According to the manufacturer, the Mio smart bracelet’s proprietary algorithms estimate EE using HR, active calories, and basal metabolic rate (BMR) data. Hence, it was expected that the EE values reported by the device would be more accurate; instead, they showed a large bias in most of the activities. This was probably due to the effects of the different physical activity levels and the exercise intensities. Therefore, the results should be viewed with caution when this device is used to measure EE.

Negative correlations were found in the validity tests for HR and EE measurement in some participants. However, analysis of data from repeated measurements showed good consistency of the results. Considering that the majority of the negative correlations were observed in high-intensity tests, we concluded that the device is unstable during high-intensity activity and that this was a significant contributing factor.

In our study, we found that the reliability of the left Mio for EE measurement was relatively poor compared with that of the right Mio. Furthermore, all participants were right-handed. Thus, we concluded that the Mio smart bracelet could measure EE accurately when worn on the wrist of the dominant hand. Since the dominant arm is stronger and used more often than the non-dominant arm [48], the wrist selected may affect the outcome in assessing physical activity [24]. However, few studies have investigated the differences in EE measurement between smart bracelets worn on the left and right hands, which may be related to the generally poor validity of smart bracelets for measuring EE.

Many previous studies [5,16,41] have tested protocols in uniform speed progression schemes, which means that the speed was not adjusted for people with different physical activity levels. This leads to the possibility that some speed levels used in experiments have not corresponded to some of the participants’ abilities, and the notion that monitoring exercise intensity is accurate only at some specific speeds, thus discouraging the application of devices to the general public. To avoid this problem, the speed progression scheme used in this study was personalized to ensure the safety of the trial and to ensure the suitability of the smart bracelet for a variety of situations.

### 4.1. Implications

In this study, the Mio smart bracelet showed good reliability and validity in terms of HR detection. The next step should involve conducting outdoor field testing of the ability of the Mio smart bracelet to detect HR and exploring its application to people with chronic diseases. In the future, wrist-worn devices can be integrated into medical treatments, such as prescriptions for exercise for patients with type 2 diabetes, hyperlipidemia, and digestive tract tumors. However, these applications require devices to provide more accurate HR and EE data than those provided by current models. Nevertheless, the good reliability and validity of the Mio smart bracelet assessed in this study for HR measurement creates the possibility of using this device to measure additional metrics, such as blood pressure and VO_2_max.

Our findings suggest that using accurate and convenient wrist-worn devices could provide benefits to people’s fitness and in competitive sports. The real-time and efficient monitoring of exercise load could help address the needs of sportspeople for scientific exercise purposes and injury prevention.

Wearable consumer devices are constantly being improved by their respective manufacturers. The formula for measuring EE needs to be focused on improving or personalizing measurements to provide more accurate data. The algorithms used by these wearable devices to measure HR and EE are proprietary and may change without the knowledge of the general public. Therefore, those who want to use these devices in healthcare settings should always be dialectically cautious about them.

### 4.2. Limitations

This study had the following limitations. First, we tested only on a treadmill, which created a laboratory environment that was more ideal than realistic daily life environments. Second, our participants were only healthy students and did not include subgroups of people who were athletic and with health conditions. As a consequence, our results cannot be generalized to other conditions.

## 5. Conclusions

This study showed that for HR measurement, the Lexin Mio smart bracelet demonstrated good reliability and validity among people with different physical activity levels performing low-, moderate-, and high-intensity exercise on a treadmill in a laboratory. For EE measurement, the Mio smart bracelet showed good reliability and low validity. Therefore, the accuracy of the EE measurement of the smart bracelet for various physical activity levels and exercise intensities must be improved before it can be integrated into exercise interventions designed to improve the personal health of those with chronic diseases.

## Figures and Tables

**Table 1:** Physical characteristics of the participants.

Characteristics	Total	LPAL (*n* = 22)	MPAL (*n* = 23)	HPAL (*n* = 20)	*p* Value	*p* Value (LPAL vs. MPAL)	*p* Value (LPAL vs. HPAL)	*p* Value (HPAL vs. MPAL)
Male, *n* (%)	24 (36.92)	0 (0.00)	7 (30.43)	17 (85.00)	<0.001	0.005	<0.001	<0.001
Female, *n* (%)	41 (63.08)	22 (100.00)	16 (69.57)	3 (15.00)				
Age (years), Mean ± SD	22.06 ± 3.03	21.45 ± 2.54	22.09 ± 2.63	22.70 ± 3.87	0.419	0.487	0.189	0.511
Height (m), Mean ± SD	168.24 ± 7.96	162.56 ± 3.66	167.03 ± 6.10	175.88 ± 7.40	<0.001	0.013	<0.001	<0.001
Weight (kg), Mean ± SD	60.76 ± 9.71	52.97 ± 5.81	61.56 ± 8.25	68.40 ± 8.31	<0.001	<0.001	<0.001	0.004
Body mass index (kg/m^2^), Mean ± SD	21.34 ± 2.00	20.02 ± 1.83	21.98 ± 1.79	22.06 ± 1.76	<0.001	0.001	<0.001	0.879
Waist circumference (cm), Mean ± SD	73.65 ± 5.45	71.02 ± 4.11	73.61 ± 5.83	76.59 ± 4.99	0.003	0.09	0.001	0.057
Hip circumference (cm), Mean ± SD	91.83 ± 8.39	88.96 ± 10.17	92.38 ± 8.40	94.35 ± 4.91	0.106	0.168	0.038	0.435
Waist-to-hip ratio, Mean ± SD	0.81 ± 0.10	0.81 ± 0.12	0.80 ± 0.11	0.81 ± 0.04	0.966	0.851	0.948	0.803
VO_2_max, Mean ± SD	38.94 ± 7.93	32.77 ± 2.69	36.65 ± 4.72	48.35 ± 5.89	<0.001	0.006	<0.001	<0.001

VO_2_max: maximum oxygen uptake; LPAL: low physical activity level; MPAL: moderate physical activity level; HPAL: high physical activity level.

**Table 2:** Descriptive HR statistics for participants.

Group	Characteristics	Baseline	low Intensity	Moderate Intensity	High Intensity	Recovery
LPAL	Left Mio, mean (SD)	92.70 ± 10.99	97.79 ± 7.88	115.26 ± 14.65	158.34 ± 10.65	116.53 ± 12.54
	Left Mio (re-test), mean (SD)	90.11 ± 9.14	98.93 ± 12.10	114.59 ± 13.09	155.40 ± 10.22	113.87 ± 10.96
	Right Mio, mean (SD)	92.87 ± 11.24	98.60 ± 8.43	116.52 ± 12.40	157.73 ± 11.34	116.51 ± 12.54
	Right Mio (re-test), mean (SD)	89.64 ± 9.75	97.59 ± 9.27	116.54 ± 12.15	154.64 ± 12.00	114.23 ± 10.42
	Polar, mean (SD)	92.90 ± 10.62	97.23 ± 7.69	117.70 ± 13.21	158.70 ± 10.60	115.52 ± 11.98
MPAL	Left Mio, mean (SD)	83.92 ± 12.10	97.79 ± 7.88	115.26 ± 14.65	158.34 ± 10.65	116.53 ± 12.54
	Left Mio (re-test), mean (SD)	83.62 ± 11.02	90.61 ± 8.66	111.32 ± 14.87	153.58 ± 13.14	110.11 ± 12.87
	Right Mio, mean (SD)	83.87 ± 12.37	92.88 ± 15.55	114.00 ± 17.59	149.11 ± 17.63	112.49 ± 15.94
	Right Mio (re-test), mean (SD)	77.81 ± 10.24	89.55 ± 12.60	129.14 ± 15.89	160.90 ± 13.17	109.13 ± 12.51
	Polar, mean (SD)	84.22 ± 11.95	88.27 ± 11.60	112.15 ± 18.21	153.29 ± 17.20	111.04 ± 14.50
HPAL	Left Mio, mean (SD)	78.40 ± 11.08	88.50 ± 9.71	128.67 ± 13.78	158.21 ± 12.74	110.30 ± 12.00
	Left Mio (re-test), mean (SD)	74.22 ± 17.99	88.16 ± 10.72	128.33 ± 15.54	156.58 ± 13.87	108.99 ± 11.73
	Right Mio, mean (SD)	74.22 ± 17.99	88.16 ± 10.72	128.33 ± 15.54	156.58 ± 13.87	108.99 ± 11.73
	Right Mio (re-test), mean (SD)	77.81 ± 10.24	89.55 ± 12.60	129.14 ± 15.89	160.90 ± 13.17	109.13 ± 12.51
	Polar, mean (SD)	78.82 ± 11.28	87.28 ± 9.64	129.14 ± 15.54	167.45 ± 19.30	110.62 ± 12.19

Mio: Lexin Mio smart bracelet; HR: heart rate; Polar: Polar Heart Rate Monitor; LPAL: low physical activity level; MPAL: moderate physical activity level; HPAL: high physical activity level.

**Table 3:** Descriptive EE statistics for participants.

Group	Characteristics	Baseline	Low Intensity	Moderate Intensity	High Intensity	Recovery
LPAL	Left Mio (test), mean (SD)	12.45 ± 3.02	13.95 ± 3.98	18.91 ± 3.77	32.18 ± 6.07	116.53 ± 12.54
	Left Mio (re-test), mean (SD)	13.68 ± 6.30	15.86 ± 4.74	20.73 ± 6.32	34.82 ± 12.08	113.87 ± 10.96
	Right Mio (test), mean (SD)	12.41 ± 3.72	14.36 ± 5.33	19.09 ± 6.53	31.14 ± 7.64	116.51 ± 12.54
	Right Mio (re-test), mean (SD)	11.82 ± 3.13	14.05 ± 3.18	19.91 ± 5.69	31.77 ± 5.14	114.23 ± 10.42
	GAS, mean (SD)	12.41 ± 3.72	14.36 ± 5.33	19.09 ± 6.53	31.14 ± 7.64	115.52 ± 11.98
MPAL	Left Mio (test), mean (SD)	11.96 ± 4.24	13.57 ± 4.71	19.70 ± 7.55	31.96 ± 11.80	116.53 ± 12.54
	Left Mio (re-test), mean (SD)	11.43 ± 3.50	13.57 ± 4.34	20.87 ± 8.04	34.17 ± 10.83	110.11 ± 12.87
	Right Mio (test), mean (SD)	12.35 ± 4.28	14.91 ± 5.27	20.17 ± 6.12	34.17 ± 8.27	112.49 ± 15.94
	Right Mio (re-test), mean (SD)	11.87 ± 4.05	14.74 ± 4.97	20.70 ± 7.21	35.70 ± 8.96	109.13 ± 12.51
	GAS, mean (SD)	12.35 ± 4.28	14.91 ± 5.27	20.17 ± 6.12	34.17 ± 8.27	111.04 ± 14.50
HPAL	Left Mio (test), mean (SD)	9.55 ± 3.46	13.20 ± 4.77	25.60 ± 10.21	37.70 ± 19.13	110.30 ± 12.00
	Left Mio (re-test), mean (SD)	11.05 ± 3.05	14.65 ± 4.06	27.40 ± 7.97	39.55 ± 10.85	108.99 ± 11.73
	Right Mio (test), mean (SD)	11.70 ± 3.67	14.30 ± 3.31	26.85 ± 7.58	39.85 ± 10.84	108.99 ± 11.73
	Right Mio (re-test), mean (SD)	12.25 ± 3.16	14.90 ± 3.67	27.20 ± 7.31	39.40 ± 9.71	109.13 ± 12.51
	GAS, mean (SD)	11.70 ± 3.67	14.30 ± 3.31	26.85 ± 7.58	39.85 ± 10.84	110.62 ± 12.19

Mio: Lexin Mio smart bracelet; EE: energy expenditure; GAS: gas-analysis system; LPAL: low physical activity level; MPAL: moderate physical activity level; HPAL: high physical activity level.

**Table 4:** Reliability of Mio for HR.

Group	Characteristics	Baseline	Low Intensity	Moderate Intensity	High Intensity	Recovery
LPAL	Left vs. Right Mio					
	*p* value #	0.528	0.283	0.554	0.374	0.932
	Correlation (r)	0.994 **	0.914 **	0.750 **	0.961 **	0.997 **
	ICC	0.994 (0.986~0.998) ***	0.912 (0.8~0.962) ***	0.740 (0.471~0.883) ***	0.959 (0.905~0.983) ***	0.997 (0.993~0.999) ***
	MAPE	0.9 (1.13)	2.28 (2.48)	4.65 (4.83)	1.39 (1.68)	0.5 (0.63)
	Retest of Left Mio					
	*p* value #	0.168	0.543	0.784	0.111	0.115
	Correlation (r)	0.656 **	0.707 **	0.677 **	0.685 **	0.799 **
	ICC	0.645 (0.316~0.836) ***	0.646 (0.318~0.836) ***	0.684 (0.377~0.855) ***	0.684 (0.377~0.855) ***	0.791 (0.562~0.908) ***
	Retest of Right Mio					
	*p* value #	0.895	0.776	0.586	0.048 *	0.535
	Correlation (r)	0.666 **	0.630 **	0.701 **	0.448 *	0.834 **
	ICC	0.659 (0.338~0.843) ***	0.627 (0.289~0.826) **	0.700 (0.404~0.864) ***	0.448 (0.042~0.727) *	0.820 (0.616~0.921) ***
	Left vs. Right Mio					
MPAL	*p* value #	0.754	0.202	0.611	0.363	0.357
	Correlation (r)	0.998 **	0.754 **	0.946 **	0.923 **	0.982 **
	ICC	0.998 (0.996~0.999) ***	0.736 (0.471~0.879) ***	0.945 (0.875~0.976) ***	0.921 (0.824~0.966) ***	0.981 (0.957~0.992) ***
	MAPE	0.61 (0.58)	6.32 (6.28)	2.97 (3.8)	2.89 (3.63)	1.67 (2.33)
	Retest of Left Mio					
	*p* value #	0.597	1	0.531	0.315	0.979
	Correlation (r)	0.585 **	0.697 **	0.462 *	0.691 **	0.591 **
	ICC	0.583 (0.233~0.799) **	0.654 (0.339~0.837) ***	0.652 (0.336~0.836) ***	0.652 (0.336~0.836) ***	0.576 (0.224~0.795) **
	Retest of Right Mio					
	*p* value #	0.776	0.591	0.426	0.169	0.396
	Correlation (r)	0.582 **	0.378	0.620 **	0.789 **	0.577 **
	ICC	0.580 (0.229~0.797) **	0.376 (−0.033~0.677) *	0.606 (0.267~0.812) **	0.768 (0.528~0.895) ***	0.570 (0.216~0.792) **
	Left vs. Right Mio					
	*p* value #	0.205	0.447	0.8	0.097	0.712
HPAL	Correlation (r)	0.992 **	0.735 **	0.845 **	−0.055	0.951 **
	ICC	0.992 (0.979~0.997) ***	0.735 (0.444~0.886) ***	0.839 (0.637~0.933) ***	−0.050 (−0.473~0.391)	0.951 (0.88~0.98) ***
	MAPE	1.29 (1.17)	4.65 (6.11)	3.67 (6.07)	6.94 (6.98)	2.26 (2.57)
	Retest of Left Mio					
	*p* value #	0.141	0.266	0.478	0.679	0.97
	Correlation (r)	0.124	0.532 *	0.795 **	0.702 **	0.497 *
	ICC	0.111 (−0.338~0.519)	0.530 (0.126~0.783) **	0.699 (0.382~0.869) ***	0.699 (0.382~0.869) ***	0.497 (0.081~0.765) *
	Retest of Right Mio					
	*p* value #	0.591	0.486	0.826	0.876	0.428
	Correlation (r)	0.556 *	0.697 **	0.813 **	−0.272	0.577 **
	ICC	0.553 (0.158~0.796) **	0.673 (0.338~0.856) ***	0.812 (0.585~0.921) ***	−0.253 (−0.618~0.202)	0.577 (0.192~0.808) **

*: *p* < 0.05; **: *p* < 0.01; ***: *p* < 0.001; *p*-value #: the *p* value of *t*-test; Mio: Lexin Mio smart bracelet; HR: heart rate; LPAL: low physical activity level; MPAL: moderate physical activity level; HPAL: high physical activity level; Correlation (r): Pearson’s correlation coefficient; ICC: intraclass correlation coefficient; MAPE: mean absolute percentage error.

**Table 5:** Reliability of Mio for EE.

Group	Characteristics	Baseline	Low Intensity	Moderate Intensity	High Intensity	Recovery
LPAL	Left vs. Right Mio					
	*p* value #	0.943	0.676	0.86	0.577	0.924
	Correlation (r)	0.639 **	0.559 **	0.693 **	0.217	0.546 **
	ICC	0.625 (0.286~0.825) ***	0.536 (0.158~0.777) **	0.600 (0.249~0.812) ***	0.211 (−0.221~0.575)	0.535 (0.156~0.776) **
	MAPE	19.58 (15.17)	20.41 (18)	16.77 (13.37)	20.68 (30.45)	16.26 (15.46)
	Retest of Left Mio					
	*p* value #	0.364	0.105	0.23	0.366	0.104
	Correlation (r)	0.273	0.277	0.139	0.025	0.402
	ICC	0.213 (−0.22~0.575)	0.273 (−0.158~0.617)	0.122 (−0.306~0.51)	0.020 (−0.396~0.43)	0.330 (−0.096~0.654)
	Retest of Right Mio					
	*p* value #	0.371	0.74	0.217	0.602	0.524
	Correlation (r)	0.620 **	0.554 **	0.887 **	0.674 **	0.780 **
	ICC	0.611 (0.264~0.818) **	0.488(0.094~0.75) **	0.879 (0.731~0.948) ***	0.624 (0.284~0.825) ***	0.773 (0.529~0.899)***
	Left vs. Right Mio					
MPAL	*p* value #	0.609	0.193	0.734	0.287	0.806
	Correlation (r)	0.640 **	0.540 **	0.544 **	0.578 **	0.694 **
	ICC	0.640 (0.318~0.83) ***	0.537 (0.169~0.773) **	0.532 (0.162~0.771) **	0.543 (0.178~0.777) **	0.683 (0.385~0.852) ***
	MAPE	21.35 (16.22)	20.81 (20.54)	19.59 (19.88)	13.99 (11.54)	21.82 (25.93)
	Retest of Left Mio					
	*p* value #	0.895	0.776	0.586	0.048 *	0.535
	Correlation (r)	0.283	0.242	0.357	0.586 **	0.600 **
	ICC	0.278 (−0.142~0.614)	0.241(−0.181~0.588)	0.357 (−0.056~0.665) *	0.584 (0.234~0.799) **	0.559 (0.2~0.786) **
	Retest of Right Mio					
	*p* value #	0.612	0.793	0.684	0.243	0.671
	Correlation (r)	0.428 *	0.813 **	0.597 **	0.754 **	0.490 *
	ICC	0.427 (0.027~0.709) *	0.812 (0.606~0.916) ***	0.589 (0.242~0.802) **	0.751 (0.498~0.886) ***	0.479 (0.093~0.74) **
	Left vs. Right Mio					
	*p* value #	0.017	0.398	0.552	0.635	0.539
HPAL	Correlation (r)	0.466 *	0.046	0.495 *	0.209	0.525 *
	ICC	0.465 (0.04~0.747) *	0.043 (−0.397~0.467)	0.474 (0.051~0.752) *	0.180 (−0.275~0.568)	0.504 (0.091~0.769) *
	MAPE	26.59 (26.01)	21.01 (16.86)	22.6 (29.53)	24.27 (28.89)	30.63 (41.26)
	Retest of Left Mio					
	*p* value #	0.36	0.881	0.877	0.49	0.628
	Correlation (r)	0.102	0.186	0.269	0.23	0.078
	ICC	0.101 (−0.347~0.512)	0.183 (−0.271~0.571)	0.261 (−0.193~0.624)	0.198 (−0.257~0.581)	0.075 (−0.37~0.492)
	Retest of Right Mio					
	*p* value #	0.661	0.276	0.998	0.277	0.565
	Correlation (r)	0.138	0.419	0.557 *	0.231	0.09
	ICC	0.137 (−0.315~0.538)	0.416 (−0.02~0.72) *	0.556 (0.162~0.797) **	0.230 (−0.226~0.603)	0.066 (−0.378~0.485)

*: *p* < 0.05; **: *p* < 0.01; ***: *p* < 0.001; *p*-value #: the *p* value of *t*-test; Mio: Lexin Mio smart bracelet; EE: Energy Expenditure; LPAL: low physical activity level; MPAL: moderate physical activity level; HPAL: high physical activity level; Correlation (r): Pearson’s correlation coefficient; ICC: intraclass correlation coefficient; MAPE: mean absolute percentage error.

**Table 6:** Validity of Mio versus Polar.

Group	Characteristics	Baseline	Low Intensity	Moderate Intensity	High Intensity	Recovery
LPAL	Left Mio vs. Polar					
	*p* value #	0.492	0.188	0.213	0.827	0.155
	Correlation (r)	0.993 **	0.969 **	0.800 **	0.738 **	0.967 **
	ICC	0.992 (0.982~0.997) ***	0.969 (0.926~0.987)***	0.796 (0.571~0.91) ***	0.738 (0.467~0.882) ***	0.966 (0.92~0.986) ***
	MAPE	1.06 (0.95)	1.66 (0.98)	4.22 (9.04)	3.05 (3.3)	2.21 (2.25)
	Right Mio vs. Polar					
	*p* value #	0.891	0.07	0.174	0.597	0.175
	Correlation (r)	0.996 **	0.916 **	0.955 **	0.704 **	0.964 **
	ICC	0.994 (0.986~0.998) ***	0.913 (0.802~0.963) ***	0.953 (0.89~0.98) ***	0.702 (0.408~0.865) ***	0.963 (0.914~0.985) ***
	MAPE	1 (0.66)	2.49 (2.17)	2.25 (2.17)	3.31 (4.14)	2.15 (2.42)
MPAL	Left Mio vs. Polar					
	*p* value #	0.307	0.166	0.203	0.026	0.333
	Correlation (r)	0.993 **	0.877 **	0.972 **	0.808 **	0.971 **
	ICC	0.993 (0.984~0.997) ***	0.875 (0.728~0.945) ***	0.971 (0.934~0.988) ***	0.805 (0.594~0.912) ***	0.965 (0.919~0.985) ***
	MAPE	1.26 (1.04)	5.25 (3.72)	2.63 (2.53)	5.38 (6.26)	2.51 (2.85)
	Right Mio vs. Polar					
	*p* value #	0.256	0.051	0.147	0.094	0.051
	Correlation (r)	0.993 **	0.727 **	0.946 **	0.784 **	0.980 **
	ICC	0.993 (0.983~0.997) ***	0.697 (0.407~0.859) ***	0.945 (0.876~0.976) ***	0.783 (0.555~0.902) ***	0.976 (0.943~0.99) ***
	MAPE	1.32 (1.08)	7.55 (6.15)	3.31 (3.49)	4.94 (6.04)	2.2 (1.56)
HPAL	Left Mio vs. Polar					
	*p* value #	0.446	0.088	0.577	0.096	0.959
	Correlation (r)	0.987 **	0.655 **	0.622 **	0.737 **	0.757 **
	ICC	0.987 (0.967~0.995) ***	0.653 (0.308~0.847) ***	0.611 (0.243~0.826) **	0.734 (0.441~0.885) ***	0.756 (0.481~0.896) ***
	MAPE	1.46 (2)	5.83 (4.57)	5.91 (6.84)	3.85 (3.92)	5.48 (4.86)
	Right Mio vs. Polar					
	*p* value	0.701	<0.001	0.411	0.362	0.893
	Correlation (r)	0.994 **	0.977 **	0.740 **	−0.21	0.833 **
	ICC	0.993 (0.983~0.997) ***	0.975 (0.938~0.99) ***	0.739 (0.45~0.888) ***	−0.199 (−0.582~0.256)	0.833 (0.626~0.93) ***
	MAPE	1.16 (1.22)	1.84 (1.46)	5.08 (8.05)	7.05(7.01)	4.49 (3.87)

**: *p* < 0.01; ***: *p* < 0.001; *p*-value #: the *p* value of *t*-test; Mio: Lexin Mio smart bracelet; Polar: Polar Heart Rate Monitor; LPAL: low physical activity level; MPAL: moderate physical activity level; HPAL: high physical activity level; Correlation (r): Pearson’s correlation coefficient; ICC: intraclass correlation coefficient; MAPE: mean absolute percentage error.

**Table 7:** Validity of Mio versus GAS.

Group	Characteristics	Baseline	Low Intensity	Moderate Intensity	High Intensity	Recovery
LPAL	Left Mio vs. GAS					
	*p* value #	<0.001	<0.001	<0.001	<0.001	<0.001
	Correlation (r)	0.342	0.198	0.782 **	0.165	0.111
	ICC	0.181 (−0.251~0.553)	0.096 (−0.33~0.49)	0.780 (0.541~0.902) ***	0.133 (−0.296~0.518)	0.058 (−0.364~0.46)
	MAPE	19.06 (14.75)	20.35 (27.08)	10.37(9.51)	13.42 (8.62)	18.46 (27.2)
	Right Mio vs. GAS					
	*p* value #	<0.001	<0.001	<0.001	<0.001	<0.001
	Correlation (r)	0.424 *	0.334	0.675 **	0.569 **	0.624 **
	ICC	0.186 (−0.246~0.557)	0.124 (−0.305~0.511)	0.564 (0.196~0.792) **	0.395 (−0.022~0.695) *	0.273 (−0.158~0.617)
	MAPE	24.52 (21.41)	27.52 (37.16)	19.13 (12.37)	18.43 (30)	19.16 (24.27)
MPAL	Left Mio vs. GAS					
	*p* value #	<0.001	<0.001	0.003	0.326	<0.001
	Correlation (r)	0.166	0.466 *	0.652 **	0.677 **	0.620 **
	ICC	0.181 (−0.251~0.553)	0.096 (−0.33~0.49)	0.646 (0.326~0.833) ***	0.611 (0.274~0.814) ***	0.330 (−0.086~0.648)
	MAPE	25.39 (28.61)	24.84 (18.4)	26(23.2)	22.08(23.77)	32.42 (35.27)
	Right Mio vs. GAS					
	*p* value #	<0.001	<0.001	<0.001	0.016	<0.001
	Correlation (r)	0.288	0.432 *	0.739 **	0.557 **	0.646 **
	ICC	0.140 (−0.28~0.515)	0.230 (−0.193~0.58)	0.738 (0.475~0.88) ***	0.554 (0.193~0.783) **	0.399 (−0.007~0.691) *
	MAPE	30.08 (21.07)	27.46 (26.97)	18.37 (17.21)	16.4(9.94)	26.12 (34.85)
HPAL	Left Mio vs. GAS					
	*p* value #	<0.001	<0.001	0.368	0.204	<0.001
	Correlation (r)	0.093	0.093	0.42	−0.014	0.402
	ICC	0.181 (−0.251~0.553)	0.096 (−0.33~0.49)	0.418 (−0.018~0.721) *	−0.011 (−0.442~0.424)	0.275 (−0.179~0.633)
	MAPE	30.27 (31.96)	28.85 (12.25)	25.57 (15.04)	28.71 (20.06)	29.1 (19.6)
	Right Mio vs. GAS					
	*p* value	<0.001	<0.001	0.607	0.198	0.001
	Correlation (r)	0.289	0.227	0.583 **	0.067	0.182
	ICC	0.199 (−0.256~0.582)	0.199 (−0.256~0.582)	0.571 (0.184~0.805) **	0.067 (−0.377~0.486)	0.099 (−0.349~0.51)
	MAPE	25.39 (25.61)	19.64 (16.07)	21.88(19.53)	24.7 (27.79)	30.33 (31.71)

*: *p* < 0.05; **: *p* < 0.01; ***: *p* < 0.001; *p*-value #: the *p* value of *t*-test; Mio: Lexin Mio smart bracelet; GAS: gas-analysis system; LPAL: low physical activity level; MPAL: moderate physical activity level; HPAL: high physical activity level; Correlation (r): Pearson’s correlation coefficient; ICC: intraclass correlation coefficient; MAPE: mean absolute percentage error.

## Data Availability

The data presented in this study are available on request from the corresponding author. The data are not publicly available due to privacy restrictions.

## Abbreviations

Mio	Mio Smart Bracelet
HR	heart rate
EE	energy expenditure
Polar	Polar H10 heart rate band
GAS	gas-analysis system
ICC	intraclass correlation coefficient
LOA	limits of agreement
MAPE	mean absolute percentage error
MD	mean difference
ECG	electrocardiography
RPE	The Borg Rating of Perceived Exertion
PAR-Q	physical activity readiness questionnaires
IPAQ	International Physical Activity Questionnaires
VCO_2_	carbon dioxide production
VO_2_	oxygen consumption
VO_2_max	maximal oxygen uptake
LPAL	low physical activity level
MPAL	moderate physical activity level
HPAL	high physical activity level
